# Subnetwork selection in deep cortical layers is mediated by beta-oscillation dependent firing

**DOI:** 10.3389/fnsys.2013.00025

**Published:** 2013-06-24

**Authors:** Thilo Womelsdorf, Stephanie Westendorff, Salva Ardid

**Affiliations:** Department of Biology, Centre for Vision Research, York UniversityToronto, ON, Canada

Even the simplest tasks in our everyday life depend on the activity in multiple brain areas that are coordinated in large-scale brain networks (Sporns, 2011). These networks restructure the information flow in the brain on fast time scales whenever we re-focus our attention on novel tasks or initiate novel movements to interact with our environment. This restructuring of information flow is implemented in cortical circuits by functionally changing the identity and composition of cells in the output layers that connect to other long-distant network nodes. Recent anatomical evidence has begun to show that these output cells form highly specific, segregated subnetworks (Krook-Magnuson et al., 2012). Cells within a subnetwork more likely interconnect with each other and share distant projection targets, avoiding interactions with other cells that project elsewhere.

**Figure 1:**
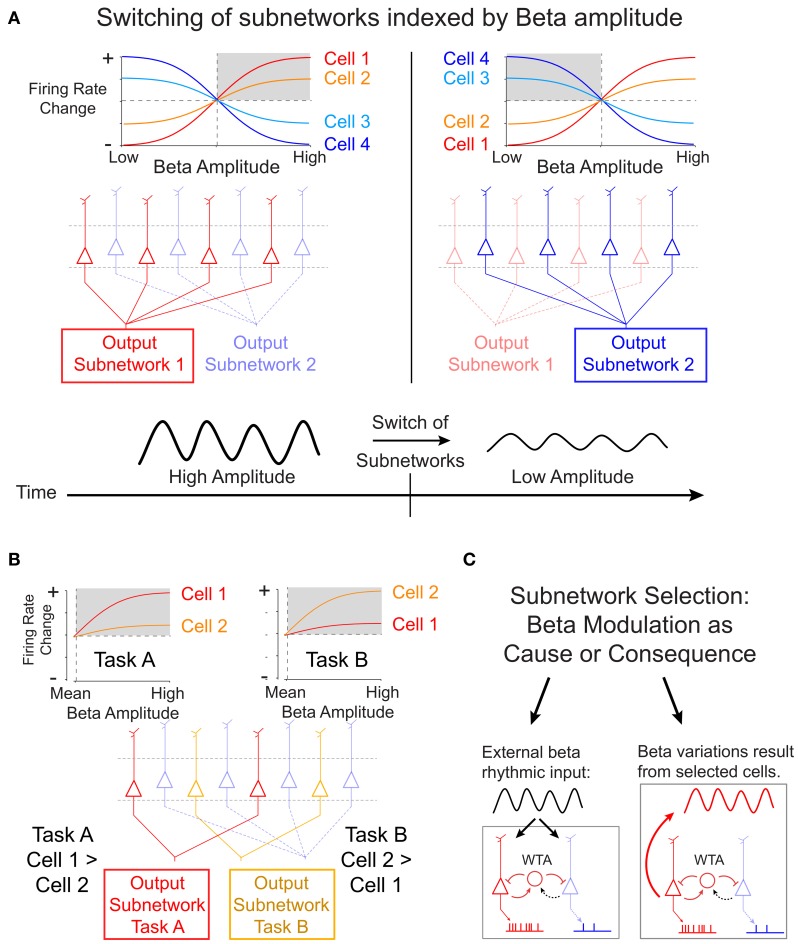
**Proposed scheme of subnetwork participation of deep layer cells by amplitude variations of beta oscillations. (A)** Canolty et al. (2012) have shown that in deep cortical layers the firing rate of single cells show a highly robust sigmoidal relation to the amplitude of beta oscillations in the local field potential. Some cells (cell 1 and 2) fire strongest during high beta amplitudes (gray shading, *left panel*), while other cells (cell 3 and 4) fire strongest during suppressed beta amplitudes (gray shading, *right panel*). The amplitude-to-rate mapping is consistent across recording sessions, suggesting that a high beta amplitude cortical state (*left panels*) indexes the activation of a selected subnetwork of deep layer cells. When beta amplitudes change over time, e.g., during the planning and execution of a movement, the subnetwork of cells with high firing switches (*right panels*). **(B)** In addition to the overall state-change, the slope of the amplitude-to-rate mapping switches reliably and reversibly when subjects engage in different tasks. This finding shows that the rank-ordering of a subset of cells according to their firing rate switches with the specific task. This task-specific re-ordering of cells could signify the formation of task-specific subnetwork (subnetwork for task A and task B in the example sketch). **(C)** The selection of subnetworks illustrated in **(A)** and **(B)** could be realized by a recurrent winner-take-all (WTA) circuit in deep cortical layers. It is open, however, whether subnetwork selection is mechanistically caused by beta rhythmic input to deep layer cells (*left panel*), or whether beta rhythmic modulation follows from cell activity after they have been selected by task- and state-specific inputs (*right panel*).

It remains unknown, however, how cells of the same deep layer subnetwork are selected when task demands change (Douglas and Martin, 2004). Results from a recent study by Canolty et al. (2012) suggest an interesting possibility to resolve this context-dependent output selection by showing that the composition of cells that fire together and in phase in the beta cycle can be inferred from the strength of local beta rhythmic modulation. Canolty and colleagues recorded the firing of cells in deep layer motor cortex of primates engaged in either of two tasks that required moving a cursor between visual stimuli manually (with their hands), or through brain activity (brain control). During these tasks, the majority of cells in motor cortex fire systematically at particular phases of a beta oscillation that reduces its amplitudes when actual or brain controlled movements are planned and executed. Canolty and colleagues show that beta synchronous firing rates of individual cells increase or decrease in close correspondence to increases or decreases in the amplitude of beta oscillations. This cell-specific mapping between firing rates and beta amplitudes was highly stable for single cells across multiple recording sessions, but it varied for a large subset of cells between different tasks in a reversible and reliable manner. These findings have potentially wide implications for our understanding of the mechanisms of rapid sub-network selection. Figure 1A illustrates the cell-specific mapping between firing rates and beta amplitudes and how a particular beta rhythmic state in a local circuit could signify which cells fire and are therefore selected into the currently active subnetwork. When the beta amplitude of the local field potentials surrounding the cell changes over time, for example when it is reduced during movement initiation, the subnetwork of cells that fired during high beta states dissipates and the circuit switches on cells that prefer firing at low beta amplitudes (Figure 1A). Furthermore, Canolty et al. showed that for one third of beta-modulated cells, the rank ordering of firing rates varied systematically during different tasks. As shown in Figure 1B, this task specificity of reliable beta-to-rate mapping could underlie the flexible and fast selection of task-specific subnetworks.

The task- and cell- specific mapping between beta amplitude and firing rate reveals a statistically robust relationship of two, in principle, independent signals. As suggested by Canolty and colleagues, this relationship would allow activating brain circuits by changing the beta rhythmic temporal structure—independently from targeting the firing rate of neurons explicitly. Such a mechanism assumes that the beta-rhythm is causal of the firing rate changes. Specifically, as indicated in Figure 1C (*left panel*), external beta rhythmic input may act as the trigger to entrain synaptic activity of cells in target areas that are resonant to beta rhythmic fluctuations of input. Stronger rhythmicity of beta rhythmic input will thereby select cells according to their beta-specific sensitivity and possibly independent of their overall level of excitation (Akam and Kullmann, 2010). According to this scenario, a change of beta rhythmic activity would serve as a true switch of a local network by causally modulating firing rates. Future studies, using stimulation techniques, will be necessary to test the prediction of beta rhythmicity being causally involved in task-specific subnetwork selection.

An alternative possibility is that varying beta amplitudes within a local circuit may not cause the switch of subnetworks, but may rather reflect the consequence of the switch itself (Figure 1C, *right panel*). According to this assumption, beta amplitudes may derive from the intrinsic properties of the cells that are selected, including e.g., beta rhythmic, intrinsic burst firing. Thus, the actual switch of active cells would follow from mechanisms that only indirectly relate to the rhythm generating mechanisms. For example, in computational firing rate models of randomly connected networks, such switches of subnetworks can be achieved by locally biasing the balance of excitation and inhibition, such that selected cells will be released from inhibition and will maintain their selectively activated state via the di-synaptic inhibition of non-selected cells (Vogels and Abbott, 2009).

Whatever the actual mechanism that causes a local circuit to switch the subnetworks of cells in deep cortical layers, the finding of a reliable mapping between beta amplitude and firing rate in the majority of deep layer cells in motor cortex critically extends our perspective of the working principles of brain activity. The results by Canolty et al. show that brain signals (1) at the local scale of cell (firing), (2) at the meso-scale of circuits (beta entrainment), and (3) at macro-scales comprising long-distant networks (inter-areal beta-coherence, not discussed here), combine together in systematic ways to subserve the larger goal to establish functional networks that can flexibly switch according to rapidly varying task demands. This “cross-level” relation of activity is only recently moving into the focus of scientific scrutiny. The discussed study and its broad analysis of available neuronal signals from all three levels of neuronal dynamics points the right way into this direction, and promises to critically advance our understanding of how external factors like specific reach movements, or the shifting of attention are implemented by the dynamics of local circuits and their dynamic interplay with larger functional brain networks.
